# Growth and spread of manufacturing productivity across regions in India

**DOI:** 10.1186/2193-1801-2-53

**Published:** 2013-02-15

**Authors:** Suresh M Babu, Rajesh Raj S Natarajan

**Affiliations:** 1 Department of Humanities and Social Sciences, Indian Institute of Technology Madras, Chennai, India; 2 Centre for Multi-Disciplinary Development Research, Dharwad, Karnataka India

**Keywords:** Growth, Productivity, Manufacturing, Reforms, Indian states, Convergence

## Abstract

**Abstract:**

An expected outcome of economic reforms in India is enhanced pace of industrialization with manufacturing sector playing a crucial role by increasing its share in output via higher investments and increased productivity. This process of industrialization was also expected to usher in possibilities for the slow growing states to catch up with the fast growing ones. This paper assesses the extent of regional manufacturing performance in India by analyzing the trends in labour and total factor productivity for the organized manufacturing sector of 15 major Indian states. Data Envelopment Analysis is used to compute Malmquist total factor productivity index and its components. The results indicate that labour productivity diverges in the reform era and its growth and TFPG follow more or less a similar pattern. The study also finds that growth in productivity vary considerably across states and this variation in productivity growth can be explained, to a great extent, by differences in infrastructural development at the regional level.

**JEL Classification:**

D24, O47, R11

The trajectory of manufacturing growth in India has been a subject of scrutiny and intense debates.^a^ Equally important has been the attempts to locate the proximate and ultimate sources of its growth. Changes in policy environment, ability to attract factor inputs and its efficient utilization have been central to such analysis. However, majority of such studies has examined economy wide trends or industry wide patterns.^b^ As manufacturing activity in India has region specific characteristics and is subjected to a number of state level legislations, regional analysis assumes importance. However, only limited attempts exist on regional industrial growth in India, especially in the wake of changes in economic policies since 1991.^c^

The nineties represent a paradigm shift in India’s economic policy making. The period witnessed significant changes in trade and industrial policies opening more avenues for private initiatives in the economy signaling a departure from the state-directed planned industrialization. The policy changes involved a concerted effort to integrate India with the rest of the world, by liberalizing trade, which could be classified into two broad groups: (a) those which were intended to reduce domestic distortions and (b) those intended to ease trade with the rest of the world. The rationale for import liberalisation by way of reduction in tariff rates and a movement away from the quantitative restrictions was to bring cost reduction via lowering the prices of intermediate inputs and to enhance competitiveness of the final products. Controls via quantitative restrictions which accounted for 90 per cent of items in the pre 1991 era, decreased dramatically to 51 per cent even as early as in 1994–95 (Joshi and Little, 1996). Along with this movement away from quantitative restrictions there were also substantial reductions in the tariff rate, from 77 per cent in 1989–90 to 29 per cent in 2005–06 (Kathuria et al., 2011).

How regions adjust to trade liberalization is an important issue to address, as trade openness affects the regional industrial growth in a number of ways. First, as an outcome of trade liberalization, location of economic activities and investment flows could be directly affected. Second, increasing access to markets and inputs aided by trade integration can influence firm performance. Third, improved flow of technology and knowledge via trade liberalization can have a bearing on production efficiency, and hence industrial growth. Thus trade liberalization could lead to a situation where regions equip themselves with a set of necessary pre-conditions to tap the potential gains emanating from increased trade flows.

In light of these potential benefits that may accrue from trade liberalization, there have been some attempts to understand its impact on manufacturing performance across the Indian states. These studies can broadly be classified as follows:

- studies focusing on the implication of trade liberalization for regional industrial growth and regional inequality. These studies test for growth convergence/divergence across states and report mixed evidence with most of the studies finding that the highest rate of divergence was in the manufacturing sector (Kar and Sakthivel, 2004; Rao et al., 1999; Das and Barua, 1996).
- Another set of studies examine the evolution of spatial distribution of manufacturing employment and production across the major states in India. These studies point to the existence of considerable heterogeneity among states without any clear pattern emerging (Sen, 2009).
- A third group of studies look at the growth and productivity performance across states. These studies ascribe a major role for regional infrastructure availability, state-level institutional environment and investment climate in determining the growth of productivity. A brief review of these studies would emphasize the necessity for furthering the analysis on regional industrial growth and its determinants in India.

Ray (2002) examined the impact of reforms on efficiency and productivity in the manufacturing sector of Indian states for the period 1986–87 through 1995–96. Using data envelopment analysis, the study noted an improvement in TFPG in most of the states during the reforms period (1991–92 to 1995–96). The study showed that improvement in technical efficiency as well as faster technical progress have contributed to the observed acceleration in productivity growth. The study also found evidence of tendency towards convergence in TFP growth rate among Indian states in the reform years. Mitra et al. (2002) while analyzing the effect of infrastructure on productivity and efficiency across the Indian states found that regional disparities are still significant in India and have been increasing over time. The study advocated more public investment in infrastructure that favors the convergence of industrial productivity.

Productivity performance of manufacturing sector in 10 major Indian states for the period 1980–81 to 2000–01 was studied by Trivedi (2004) using both growth accounting (GA) and production function approach. The study found considerable inter-state differences in productivity levels and growth rates. Veeramani and Goldar (2005) examined the effect of investment climate on state-level total factor productivity in manufacturing and found that the state-level investment climate is a major determinant of productivity performance during 1980 to 2000 period. They also noticed that states with more pro-worker legislation experience lower productivity growth during the same period. In a recent study on the organized manufacturing sector in India for the period 1980–81 to 2003–04, Trivedi et al., 2011 notes significant variation in TFPG of the manufacturing sector across Indian states. The study also finds that there has been a deceleration in TFPG in the period following reforms. According to the study, it is the supply constraint in the form of technological upgradation and organizational and institutional constraints that seem to be the major factor affecting the growth of manufacturing sector. A brief review of these studies suggests that there exist limited attempts at understanding the factors that explain the productivity differential across states in India. We also find that most of these studies except Ray (2002) have not addressed the question of convergence in productivity growth across states.^d^ Moreover, these studies have considered a time period which does not extend beyond the initial phase of reforms.

Against this background the objectives of this paper are two fold: (a) it attempts to analyse the patterns of labour and total factor productivity growth in the manufacturing sector using state-level data from the Annual Survey of Industries for the period 1980/1 to 2007/8. In doing so, we also test whether productivity gaps across states are widening or shrinking (b) it also attempts to unravel the components and determinants of total factor productivity growth at the regional level.

The paper is organized as follows. Section 1 presents some evidence on industrial growth at the state level. A discussion of the methodology and data used for computing productivity growth is provided in section 2. Section 3 discusses the main results. The last section concludes.

## State-level manufacturing performance: An overview

The share of manufacturing sector in the states’ domestic product is presented in Table 1. We find that the manufacturing sector has not assumed the role of a principal contributor in states’ income over the last three decades, more or less following the national pattern. The contribution has varied between 3 and 32 percent across the states during the time period considered. The following observations emerge on the role of manufacturing sector in the last three decades: (a) some states have witnessed a marked increase in the share of manufacturing sector in state income (Gujarat, Punjab, Haryana and Bihar), (b) states like Assam, Andhra Pradesh and Rajasthan have maintained a consistent share, (c) while West Bengal and Tamil Nadu have reported a consistent decline.

**Table 1:** **Share of manufacturing sector in NSDP**

States	1980-81	1990-91	2000-01	2007-08
Andhra Pradesh	8.2	11.9	11.5	11.5
Assam	9.9	9.8	8.1	9.2
Bihar	3.3	4.7	14.5	12.4
Gujarat	17.7	24.7	24.6	26.5
Haryana	13.5	19.0	18.9	18.0
Karnataka	14.8	19.0	14.4	14.7
Kerala	10.8	11.8	11.1	8.8
Madhya Pradesh	10.2	13.2	14.2	12.1
Maharashtra	24.8	25.5	20.4	20.3
Orissa	7.8	8.4	8.8	12.7
Punjab	8.8	12.9	14.7	15.1
Rajasthan	11.5	11.6	14.0	12.4
Tamil Nadu	31.9	28.1	21.8	21.0
Uttar Pradesh	8.6	13.2	11.6	11.3
West Bengal	20.2	17.0	16.1	14.7
Mean	13.5	15.4	15.0	14.7

Source: National Accounts Statistics.

Viewed from an economy wide perspective, manufacturing activity seems to be concentrated in a few states (Table 2). The top three states (Maharashtra, Gujarat and Tamil Nadu) remain the same and the combined share of these states in gross value added remained constant (around 45 per cent) in the last 27 years. The geographical spread of industrial activity shows no significant change during the last three decades indicating that the trade liberalization policies have not been able to alter the regional dimension of industrialization. What is even more interesting is the fact that the states which have assumed the status of early movers in implementation of reforms such as Andhra Pradesh does not seem to have yielded significant outcomes. On the other hand, there seems to be a compelling need for states like West Bengal to accelerate the process of industrialization as its share witnessed a decline over time. Thus we find that manufacturing activity still continues to be concentrated in a few states despite successive changes in trade regime.

**Table 2:** **Share of states in manufacturing value added**

States	1980-81 to 1989-90	1990-91 to 1999-2000	2000-01 to 2007-08	1980-81 to 2007-08
AP	5.2	6.3	6.5	6.0
Assam	1.5	1.2	1.1	1.3
Bihar	6.5	4.5	4.0	5.0
Gujarat	10.5	11.7	14.2	12.0
Haryana	3.0	3.5	4.3	3.6
Karnataka	4.9	5.5	6.7	5.7
Kerala	2.6	2.3	1.7	2.2
MP	5.4	5.4	5.2	5.3
Maharashtra	23.4	22.3	21.3	22.4
Orissa	1.8	1.8	2.1	1.9
Punjab	3.1	3.5	2.8	3.1
Rajasthan	2.4	2.9	3.0	2.8
Tamil Nadu	10.4	10.6	9.5	10.2
Uttar Pradesh	6.9	8.2	7.1	7.4
West Bengal	8.4	5.0	3.7	5.8
Share of top three states	44.3	44.5	45.0	44.6

Source: Annual Survey of Industries.

Note: We use gross value added in 1993/94 prices. Figures are averages in percent.

To provide further insights on concentration of manufacturing activity, the study also examines regional dispersal of employment and investments. The combined share of top three states in total manufacturing employment in the economy has increased slightly over time (Table 3). Seven out of 15 states have witnessed erosion in their share in total manufacturing employment. In terms of gainers and losers in employment, the study finds Tamil Nadu increasing its share substantially while Uttar Pradesh, Bihar and West Bengal witnessing decline with West Bengal recording maximum decline. A one to one relationship between employment shares and output share holds good for majority of the states except Gujarat, Kerala, Tamil Nadu and Uttar Pradesh.

**Table 3:** **Share of states in employment 1980–81 to 2007–08**

States	1980-81 to 1989-90	1990-91 to 1999-2000	2000-01 to 2007-08	1980-81 to 2007-08
AP	9.6	10.9	10.8	10.4
Assam	1.6	1.5	1.4	1.5
Bihar	4.6	3.5	2.3	3.6
Gujarat	9.9	9.3	9.3	9.5
Haryana	2.8	3.4	4.2	3.4
Karnataka	5.0	5.7	6.5	5.7
Kerala	3.2	3.7	3.7	3.6
MP	4.3	4.5	3.6	4.2
Maharashtra	17.3	15.5	14.0	15.7
Orissa	1.6	1.7	1.6	1.6
Punjab	3.7	4.1	4.7	4.1
Rajasthan	2.3	2.7	3.1	2.7
Tamil Nadu	11.4	13.2	15.2	13.1
Uttar Pradesh	9.2	8.3	7.6	8.4
West Bengal	11.8	8.6	6.2	9.1

Source: Annual Survey of Industries.

Note: Figures are averages in percent.

Improving investment climate at the state level has occupied a prime place in the economic reforms package. Earlier evidence on output and employment shares seems to be corroborated by the evidence on investment. Two states namely Maharashtra and Gujarat accounted for nearly 34 per cent of total actual investments in the manufacturing sector for the period prior to reforms (Table 4). In the era of policy reforms, the same two states have been able to maintain their high shares. The reforms package intended to unleash centrifugal forces to spread the investments to new destinations does not seem to have yielded the desired results. In fact, Uttar Pradesh and Madhya Pradesh which had substantial shares in investment in the earlier period seem to have lost out to Gujarat and Andhra Pradesh.

**Table 4:** **Share of states in total investment 1980–81 to 2007–08**

States	1980-81 to 1989-90	1990-91 to 1999-2000	2000-01 to 2007-08	1980-81 to 2007-08
AP	6.8	7.1	10.3	8.0
Assam	2.1	0.3	2.4	1.1
Bihar	1.7	1.7	2.2	1.8
Gujarat	10.2	19.1	19.7	18.3
Haryana	2.7	3.5	4.5	3.8
Karnataka	4.7	7.3	7.1	7.0
Kerala	1.9	1.2	0.2	1.0
MP	8.0	5.8	2.7	5.1
Maharashtra	23.6	16.9	11.3	15.8
Orissa	8.3	1.3	13.4	5.8
Punjab	2.9	2.3	3.2	2.6
Rajasthan	4.1	5.5	−2.7	2.8
Tamil Nadu	9.8	9.5	10.1	9.7
Uttar Pradesh	12.9	10.0	6.1	9.1
West Bengal	4.1	3.8	2.7	3.5

Source: Annual Survey of Industries.

Note: Figures are averages in percent.

While shift share analysis provides a relative picture of states, examining growth rates could capture temporal dimension, for which we use three indicators, growth of employment, value added and capital stock across three sub-periods (Table 5). Growth of employment presents a fluctuating trend over time. After relatively higher growth rate in the 1980s, we find a decline in employment growth during the 1990s followed by a revival post-2000-01. This picture holds good across most of the states except Bihar and West Bengal. Contrary to employment growth trends, growth of fixed capital shows a declining trend in the third sub-period compared to the first period. However it should be noted that states like Andhra Pradesh and Orissa witnessed a turn around in growth of capital stock in the third period compared to the second period. Surprisingly Maharashtra, Gujarat and Tamil Nadu major industrial investment destinations, witnessed slower growth of fixed capital stock in the post 2000 time period. It is interesting to note that in the case of value added we find a similar trend to that of employment growth. The increased growth of value added in post 2000 time period despite slower growth of investments can partly be attributed to the lag effects of investments in the earlier time period.^e^ The higher rates of value added growth of industrially less advanced states like Bihar, Orissa and Assam in the 90s and post-2000s also points to realization of some benefits from the changes in policy environment. This however, warrants further detailed examination, which we do not attempt here.

**Table 5:** **Growth of employment, gross value added and fixed capital stock**

State	Growth of employment				Growth of value added				Growth of capital stock			
	I	II	III	IV	I	II	III	IV	I	II	III	IV
AP	1.9	1.2	1.6	1.7	10.9	6.1	11.8	10.1	18.4	3.0	7.2	10.3
Assam	−0.03	0.4	2.1	0.7	14.2	−0.1	8.3	7.8	10.2	11.2	2.2	9.0
Bihar	0.3	−3.0	−0.6	−1.2	10.5	0.3	14.9	8.3	1.1	5.4	1.0	2.8
Gujarat	−1.6	1.3	4.1	1.0	6.8	9.0	11.6	9.5	9.6	14.8	4.5	10.8
Haryana	3.2	2.9	6.9	4.4	9.3	7.3	10.4	9.5	7.7	13.7	5.5	9.9
Karnataka	0.5	1.8	5.4	2.5	8.3	6.1	13.6	9.5	6.3	14.5	5.5	9.7
Kerala	0.4	2.2	1.4	1.4	6.7	5.8	2.2	5.6	5.8	8.1	0.3	5.5
MP	2.3	−0.1	2.2	1.5	9.6	4.4	11.4	8.7	7.6	7.0	5.7	7.4
Maharashtra	−2.6	0.2	1.7	−0.5	6.8	4.8	11.8	7.9	9.0	9.8	4.2	8.6
Orissa	1.2	1.1	4.7	2.2	9.9	3.2	18.3	10.2	11.6	8.0	13.9	11.7
Punjab	4.0	1.2	5.4	3.6	10.6	3.5	10.9	8.6	6.3	7.8	8.1	7.9
Rajasthan	2.5	1.7	6.2	3.4	11.6	7.1	7.0	9.3	10.4	10.8	2.6	9.0
Tamil Nadu	1.8	2.4	4.0	2.8	8.9	5.5	7.1	7.7	9.5	11.3	5.2	9.6
Uttar Pradesh	0.5	−1.4	5.3	1.1	11.3	4.5	10.3	9.1	12.3	11.8	4.3	10.7
West Bengal	−2.8	−1.8	−1.3	−2.2	2.1	1.5	7.2	3.4	6.3	6.7	3.7	6.1

Source: Authors’ computations.

Note: I, II, III and IV periods denote 1980–81 to 1990–91, 1990–91 to 2000–01, 2000–01 to 2007–08 and 1980–81 to 2007–08 respectively.

The following inferences can be drawn from the above analysis (a) dispersion of industrial activity has proceeded with a sluggish pace with the dominant states still cornering substantial shares in investments, output and employment, (b) the initial phase of 1990s, coinciding with the liberalization policies in the economy witnessed slower growth of employment and output but not investments and (c) output and employment growth picks up in the post 2000 time period across the states with less industrialized states registering high magnitudes. Given this backdrop, it would be interesting to analyse the sources of growth and its relation with trends in productivity across states to understand the possibilities of gains from reforms, if any, in accelerating productivity growth.

## Methodology and data

In view of the importance of measuring partial factor productivity, especially labor productivity, the study computes the levels and trends in both partial and total factor productivity. Labour productivity, defined as gross value added divided per person is the partial factor productivity measure used. However, caution needs to be applied when using partial factor productivity (PFP) measures as changes in input proportions can influence these measures. In a situation where capital-labour ratio follows an increasing trend, productivity of labour is overestimated and that of capital, underestimated. In this case, a change in labour productivity is merely a reflection of substituting one factor by another. Measuring total factor productivity (TFP) tries to circumvent the problem encountered in the interpretation of PFP estimates in the event of changing factor intensities. TFP is defined as the ratio of output to a weighted sum of the inputs used in the production process. It aims at decomposing changes in output due to changes in quantity of inputs used and changes in all the residual factors such as change in technology, capacity utilisation, quality of factors of production and learning by doing.

Econometric estimation of TFP growth has proceeded with two approaches on the assumption of the existence of production function – frontier and non-frontier. The crucial distinction between the frontier and non-frontier approaches lies in the very definition of the word ‘frontier’. In frontier approach aim is to find the bounding function i.e., the best obtainable positions given the inputs or the prices. A ‘cost frontier’ traces the minimum attainable cost given input prices and output and a ‘production frontier’ traces the set of maximum obtainable output for a given set of inputs and technology. The average function, on the other hand, is naturally associated with mean output and given input levels.

The TFP growth as obtained from frontier approach consists of two components - outward shifts of the production function resulting from technological progress, and technical efficiency related to the movements towards the production frontier. On the other hand, the non-frontier approach considers technological progress as a measure of TFP growth. We employ frontier approach to estimate total factor productivity in this study. TFP growth is estimated using the Malmquist productivity index. Data envelopment analysis (DEA), a non-parametric method, is used to estimate the Malmquist index. The stringent assumptions on the product market structure and weak price information could be avoided by using the Malmquist index. Moreover, a non-parametric estimation method does not require an exact specification of functional form for the underlying production function.

Charnes et al. (1978) proposed the data envelopment analysis (DEA) approach to construct a best practice frontier without specifying production technology. Unlike traditional methods that look for the average path through the middle points of a series of data, DEA looks for a best practice frontier within the data. Using a nonparametric linear programming technique, DEA constructs a production frontier from observed input–output data of the sample units. The efficiency of units is then measured in terms of how far they are from the frontier. According to Gong and Sickles (1992), DEA is more appealing than the econometric model as inefficiency is likely to be correlated with the inputs.^f^ DEA can be either input-orientated or output-orientated. In the input-orientated case, the DEA method defines the frontier by seeking the maximum possible proportional education in input usage, with output levels held constant, for each state while in the output orientated case, the DEA method seeks the maximum proportional increase in output production, with input levels held fixed. The output- and input-oriented measures provide equivalent measures of technical efficiency when constant returns to scale exist (Fare and Lovell, 1978).

The DEA approach outlined by Fare et al. (1994) is employed to construct the best practice frontier at each time period for each technology category. Comparing each state to the best-practice frontier provides a measure of its catching up in efficiency to that frontier and a measure of shift in the frontier (or technological progress). The Malmquist indexes, which measure the change in TFP, are calculated as a product of these two components.

The Malmquist productivity index^g^ is defined by using distance functions. The Malmquist TFP index measures the TFP growth change between two data points by calculating the ratio of the distances of each data point relative to a common technology. Following Fare et al. (1994), the output-oriented Malmquist TFP change index between period s (the base period) and period t (the terminal period) is given by1

where the notation *d*_0_^*s*^(*y*_*t*_, *x*_*t*_) represents the distance from the period *t* observation to the period s technology. A value of *m*_*0*_ greater than one indicates positive TFP growth from period *s* to period *t* while a value less than one indicates a TFP growth decline. Note that while the product of the efficiency change and technical change components must by definition equal the Malmquist index, they may be moving in opposite directions. For instance, a Malmquist index of 1.25 (which signals a productivity gain) could have an efficiency-change component less than one (say, 0.5) and a technical change component greater than 1 (say, 2.5).

The term inside the square bracket is the geometric mean of the shifts in technology observed in period s and period t or the frontier effect which tells us how far the efficient frontier itself has shifted over time due to the use of better technology and equipment. The term outside the square bracket measures the output-oriented measure of Farrell technical efficiency between period *s* and period *t* or the catching up effect indicating how far the industry has moved towards the efficient frontier due to the better use of technology and equipment. In other words, TFP growth can be decomposed as,

TFP Growth = Technical Efficiency Change (Catching up Effect)

× Technical Change (Frontier Effect)

As mentioned earlier, the study uses the linear programming (LP) technique known as data envelopment analysis (DEA) to calculate the distance functions.^h^ This required solving of four LPs for each state. The LPs are:

and

where y_it_ is a MXI (M x 1)vector of output quantities for the *i*-th state in the *t*-th year;

x_it_ is a KXI (K x 1) vector of input quantities for the *i*-th state in the *t*-th year;

Y_t_ is a NXM (N x M) matrix of output quantities for all N states in the *t*-th year;

X_t_ is a NXK (N x K) matrix of input quantities for all N states in the *t*-th year;

λ is a NXI (N x 1) vector of weights and ϕ is a scalar.

The Annual Survey of Industries (ASI) data for the factory sector for fifteen major states in India is used for the analysis.^i^ The period of the study covered 27 years since 1980–81. For analyzing the impact of trade reforms on TFPG in Indian manufacturing, the entire time period is divided into three sub-periods, 1980–81 to 1990–91, 1991–92 to 2000–01 and 2001–02 to 2007–08.

The study considered a single output, two-input technology for the states under investigation. The inputs are: (a) labour and (b) capital. The variables are defined as follows:

The variables for the data envelopment analysis are real value added and real capital stock at 1993–94 prices and number of persons employed. Real value added is obtained by deflating nominal value added using the wholesale price index (WPI) for manufactured products.^j^ Labour is measured as total number of persons engaged in the production activity, which include production workers as well as employees. Real capital stock is constructed by deflating gross fixed assets by WPI for machinery and machine tools.

## Results

### Productivity estimates

**Figure 1:**
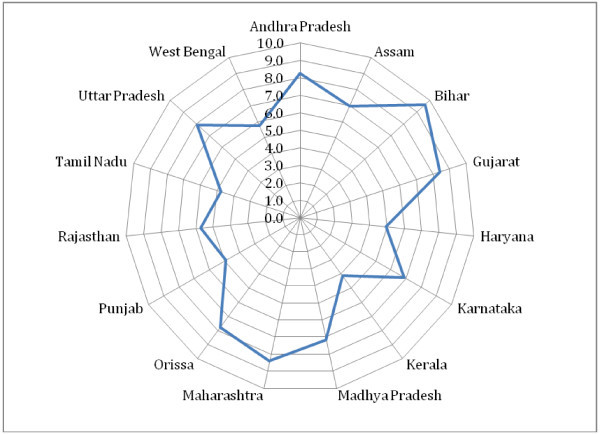
**Growth of Labour Productivity, 1980-81-2007-08.**

**Figure 2:**
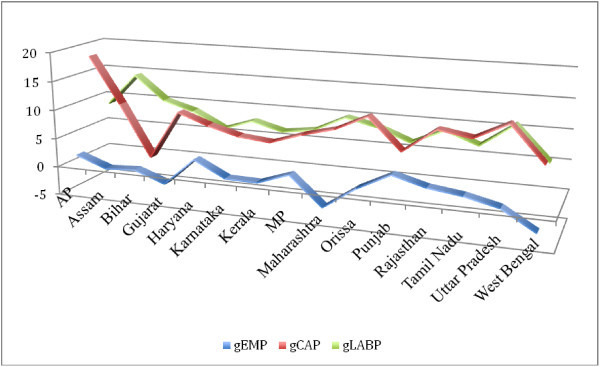
**Growth of Employment, Capital Stock and Labour Productivity: 1980–81 – 1990–91.**

**Figure 3:**
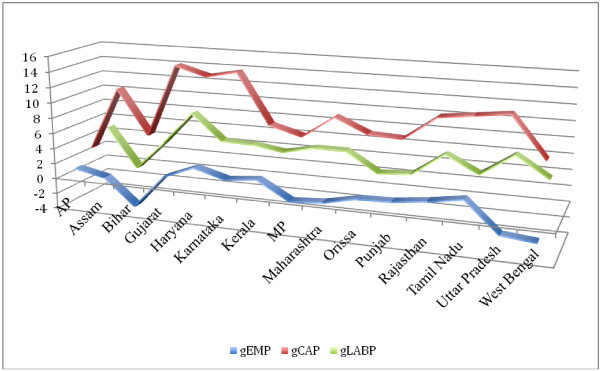
**Growth of Employment, Capital Stock and Labour Productivity: 1990–91 – 2000–01.**

**Figure 4:**
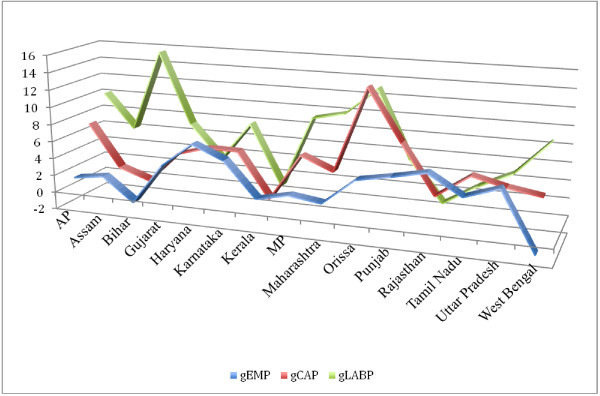
**Growth of Employment, Capital Stock and Labour Productivity: 2000–01 – 2007–08.**

We examine trends in labour productivity (LP) before proceeding to the analysis of total factor productivity growth. For the period 1980/81 to 2007/08 we find significant growth in labour productivity across states. The growth of LP is of the range between 4 and 10 per cent, with states like Bihar, Maharashtra, Andhra Pradesh and Gujarat registering highest growth rates (Figure 1). Period-wise analysis reveals that, on average, LP registers faster growth during the period 1980 /81 to 1990/91 and slower growth for the period 1991/92 to 2000/01 (Table 6). In the subsequent period, we find a revival in growth but not as high as in the 80s. We also find that the states where capital stock grew faster register faster growth in labour productivity and slower growth in employment (See Figures 2, 3 and 4).

**Table 6:** **Growth of labour productivity**

State	Growth of LP		
	1980-81 to 1990-91	1991-92 to 2000-01	2001-02 to 2007-08
Andhra Pradesh	8.8	4.8	10.1
Assam	14.3	−0.5	6.1
Bihar	10.1	3.4	15.5
Gujarat	8.6	7.7	7.2
Haryana	5.9	4.3	3.3
Karnataka	7.7	4.2	7.8
Kerala	6.3	3.6	0.8
Madhya Pradesh	7.1	4.5	9.1
Maharashtra	9.7	4.5	9.9
Orissa	8.5	2.0	13.0
Punjab	6.4	2.3	5.3
Rajasthan	8.9	5.2	0.7
Tamil Nadu	6.9	3.1	3.0
Uttar Pradesh	10.8	5.9	4.8
West Bengal	5.1	3.3	8.6

Source: Authors’ computations.

Estimates of TFPG show that for the entire period TFP has improved across all the states except for two states, Tamil Nadu and West Bengal (Table 7). We also find that the rates of growth vary across states with four states registering highest TFPG. Decomposition of TFPG into efficiency change and technical progress reveal that technical progress is the main driver in all the states. It may also be noted that only four out of fifteen states witnessed growth in efficiency and technical progress propelling TFPG. Period-wise analysis shows that in the second period there has been a slow growth of TFP, which revives in the third period indicating a fluctuating trend (Table 8). For the 80s, we find that 13 out of 15 states register a growth in TFP aided by both efficiency change and technical progress. However, the 90s present a different picture with eight out of 15 states registering decline in TFP due to both decline in efficiency and lack of technological improvement. Mainly driven by technical progress, the period after 2000 witness improvement in TFP in all the states. However, efficiency change in this time period is slower compared to the earlier periods.

**Table 7:** **Region-wise trends in TFPG and its components, 1980/1-1981/2 – 2006/7-2007/8**

State	EffCh	TechCh	TfpCh
Andhra Pradesh	1.005	1.004	1.009
Assam	0.995	1.015	1.010
Bihar	1.013	1.082	1.097
Gujarat	0.997	1.074	1.071
Haryana	0.989	1.016	1.005
Karnataka	0.996	1.031	1.026
Kerala	1.007	0.994	1.002
Madhya Pradesh	0.990	1.075	1.064
Maharashtra	1.000	1.043	1.043
Orissa	0.995	1.079	1.074
Punjab	1.002	1.009	1.011
Rajasthan	0.994	1.063	1.058
Tamil Nadu	0.988	1.000	0.988
Uttar Pradesh	0.992	1.012	1.004
West Bengal	0.980	1.006	0.985
Mean	0.996	1.033	1.029

**Note:** (a) Annual average growth rates are reported.

(b) Bihar, Madhya Pradesh and Uttar Pradesh include Jharkhand, Chattisgarh and Uttarakhand respectively.

(c) EffCh, TechCh and TfpCh denote efficiency change, technological change and total factor productivity change respectively.

**Table 8:** **Region-wise trends in TFPG and its components**

State	1980/1-1981/2 – 1989/0-1990/1			1990/1-1991/2 – 1999/0-2000/1			2000/1-2001/2 – 2006/7-2007/8		
	EffCh	TechCh	TfpCh	EffCh	TechCh	TfpCh	EffCh	TechCh	TfpCh
Andhra Pradesh	0.965	1.002	0.967	1.065	0.962	1.024	0.980	1.072	1.051
Assam	1.062	1.016	1.079	0.936	0.965	0.904	0.989	1.088	1.075
Bihar	1.020	1.088	1.110	0.988	1.046	1.033	1.041	1.127	1.173
Gujarat	0.992	1.067	1.058	1.033	1.042	1.076	0.955	1.133	1.082
Haryana	1.000	1.021	1.021	0.988	0.972	0.961	0.975	1.076	1.049
Karnataka	1.016	1.039	1.056	0.970	0.984	0.955	1.004	1.087	1.092
Kerala	1.029	0.980	1.009	1.016	0.965	0.980	0.965	1.060	1.023
Madhya Pradesh	0.989	1.082	1.070	0.999	1.042	1.042	0.976	1.112	1.085
Maharashtra	1.000	1.041	1.041	1.000	1.008	1.008	1.000	1.099	1.099
Orissa	1.003	1.085	1.088	0.974	1.046	1.019	1.015	1.119	1.136
Punjab	1.030	1.015	1.045	0.998	0.962	0.960	0.968	1.072	1.038
Rajasthan	1.001	1.084	1.086	1.007	1.036	1.044	0.967	1.074	1.038
Tamil Nadu	1.013	0.988	1.001	0.989	0.963	0.952	0.951	1.075	1.022
Uttar Pradesh	1.011	0.992	1.003	0.987	0.990	0.977	0.972	1.074	1.044
West Bengal	0.975	0.987	0.963	0.992	0.972	0.963	0.970	1.084	1.051
Mean	1.007	1.033	1.040	0.996	0.997	0.993	0.982	1.090	1.071

Note: Same as in Table 7.

Overall, we find that labour productivity growth and TFPG follow more or less a similar pattern. Our evidence also shows that growth of capital seems to have an influence on TFPG with a lag effect. This is evident from the fact that during the 90s when the economy witnessed increased investments TFPG did not register a contemporaneous increase but increased only in the post 2000 era. As pointed out by Tybout (2000), firms in reforming economies undergo an adjustment process to the changed environment in order to reap benefits from the changes which get captured in the data for later periods. The fluctuating trends in TFPG at the regional level support the existing evidence relating to the manufacturing sector economy wide (See Trivedi et al., 2011).

### Convergence of labour productivity

In an economy with initial low levels of productivity, tendency for regions to catch up and converge in the long run both in terms of output and productivity has been a matter of debate in growth literature. However, the empirical evidence for such convergence is mixed even with alternate specifications and different time periods. ^k^ Pro-market reforms in India have been implemented with an objective to improving the ‘investment climate’ in the states to attract investments to catch up with the fast growing regions. To further the argument for more doses of labour market reforms recent studies have tested for convergence between states in India and compared with another economy, which has pursued more ‘progressive’ labour market reforms, namely China. These studies^l^ have shown that across states in India labour productivity does not show any trend towards convergence.

**Figure 5:**
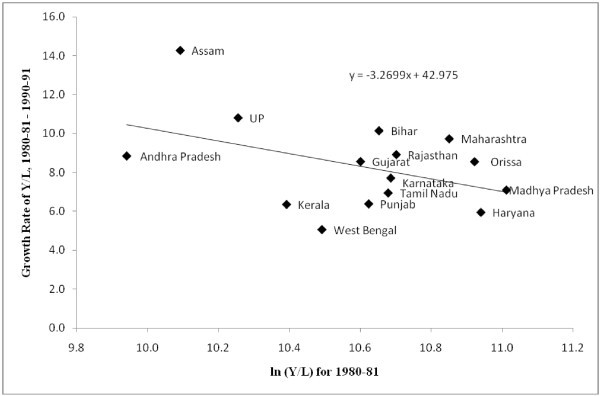
**Labour Productivity Convergence across Indian states, 1980–81 – 1990–91.**

**Figure 6:**
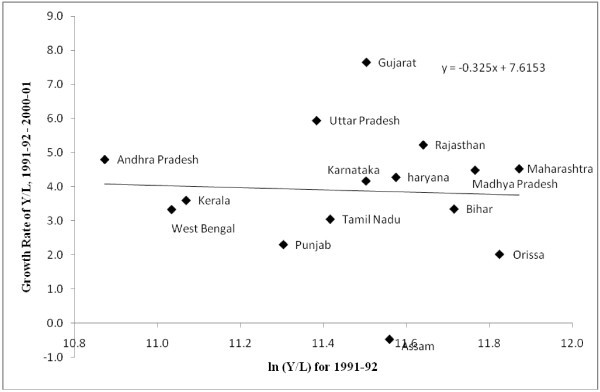
**Labour Productivity Convergence across Indian states, 1991–92 – 2000–01.**

**Figure 7:**
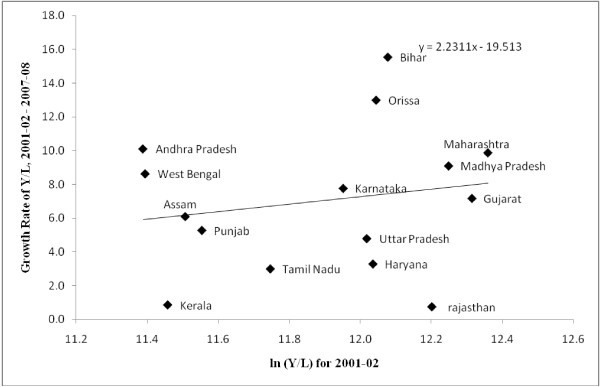
**Labour Productivity Convergence across Indian states, 2001–02 – 2007–08.**

We test for labour productivity convergence across states (Figures 5, 6 and 7). ^m^ The estimation is carried out for three sub-periods, 1980-81-1990-91, 1991-92-2000-01 and 2001-02-2007-08. The results indicate that prior to 1991, the watershed year in terms of economic reforms, labour productivity across states had a tendency to converge. Importantly the variation in labour productivity was not very high. However, the period since 1991 show a tendency towards divergence of labour productivity across regions, which further exacerbated in the post-2000 period.^n^ Despite the faster growth of labour productivity in the post-2000 period, the lack of convergence in labour productivity growth across regions demands a much closer scrutiny at the issue. Similarly, the fast growth achieved by states like Bihar needs explanation about the route to this faster growth. Collating evidence on output and employment growth it can be seen that this fast growth is due to a drastic decline in employment. Workforce shedding has raised the labour productivity without incurring substantial capital investments. This also points to the necessity for closer scrutiny on the route for convergence rather than overemphasizing the role of end results of the convergence analyses and labour market reforms.

### Factors driving productivity growth at the regional level: An econometric analysis

The above discussion suggests wide variation across the states in the rate of acceleration in productivity growth rate. It is, therefore, important to analyze the factors that account for such variation. A regression exercise is carried out to identify the determinants of TFPG at the regional level for the organized manufacturing sector. We estimated models with different specifications. The analysis is performed on a panel dataset for 15 major states for the period 1981–82 to 2007–08. The following regression function is estimated:2

Where TFPG is total factor productivity growth, the subscript s stands for state and t for time. X_st_ is a vector of state-level explanatory variables that vary across states and over time. In India, low infrastructure development has become a crucial impediment to the growth of industrial sector. The positive role of infrastructure for improving manufacturing productivity has received considerable attention in the literature (Mitra et al., 2002). It is argued that targeting public investment towards infrastructure can strongly promote convergence of industrial productivity and advance balanced regional growth. An attempt is therefore made to examine whether significant differences in manufacturing productivity as observed across Indian states can be explained by differences in infrastructures endowments. Drawing from the literature, we have identified three dimensions of infrastructure development at the regional level; power, transport and telecommunications. Two proxies are identified to represent power availability: (a) gross power generation per capita (ChELECGEN) and (b) industry share in total power sales in the state (INDSALE). Road density (RDDEN) is used to represent availability of transport and telephone density (TELDEN) for spread of telecommunications across the states. As a social infrastructure indicator, we also include literacy rate (LITERACY) in our empirical exercise. ^o^ Five different regression models are estimated as shown in Table 9. Models 1 and 2 differ with regard to the inclusion of ChELECGEN as a variable in the latter. In Model 3, we introduce RDDEN while TELDEN and LITERACY also enter as explanatory variables in Model 4. Model 5 estimates the regression function without ChELECGEN. ^p^

**Table 9:** **Determinants of TFPG at the regional level: regression results**

Dep. Variable: Total factor productivity growth					
Variables	Model1	Model 2	Model 3	Model 4	Model 5
INDSALE	0.159* (0.080)	0.165* (0.080)	0.162* (0.079)	0.255* (0.090)	0.257* (0.090)
ChELECGEN	-	0.080* (0.048)	0.074* (0.043)	0.068@ (0.045)	-
RDDEN	-	-	0.403@ (0.268)	0.406@ (0.271)	0.411@ (0.271)
TELDEN	-	-	-	1.172* (0.525)	1.255* (0.510)
LITERACY	-	-	-	2.440* (1.118)	2.513* (1.112)
Constant	−0.024 (0.032)	−0.031 (0.032)	−0.042 (0.035)	−0.160* (0.057)	−0.160* (0.057)
R Squared	0.02	0.02	0.06	0.07	0.07
N	405	405	405	405	405

Note: INDSALE stands for the share of industry in total electricity sales; ChELECGEN stands for changes in electricity generation; RDDEN denotes road density; and TELDEN denotes telephone density.

@ Significant between 10 and 15 per cent level.

Our results clearly show that infrastructure availability is a key variable influencing regional manufacturing productivity growth in India. The coefficients of INDSALE, ChELECGEN, RDDEN and TELDEN are positive and significant in all specifications indicating that all dimensions of infrastructural development are important in improving manufacturing productivity at the state level.^q^ The positive and significant coefficient of LITERACY suggests that improving the human capital base of workers in the sector could be a source of big productivity improvements in manufacturing industries. With regard to power availability, the positive coefficients of ChELECGEN and INDSALE suggests that along with improving the power generation, enhancing the availability of power to the industrial sector is also an important factor in increasing total factor productivity. Similarly, improved road connectivity and better telecommunication facilities also seem to make significant impact on the productivity performance of the sector in Indian states. Our analysis thus confirms that differences in manufacturing productivity growth across states can be explained, to a large extent, by differences in levels of infrastructural development.

## Conclusions

Our analysis shows that dispersion of industrial activity has proceeded with a sluggish pace with the dominant states still cornering substantial shares in investments, output and employment despite successive changes in policy regime. We find that output and employment growth in the manufacturing sector registered a slow growth in the 1990s, the period that coincides with economic reforms in India. However, the period since 2000 witnessed significant revival of manufacturing activities in India with improved performance in value addition and employment generation especially in the less industrialized states. Concomitant to this, the period also witnessed significant improvement in productivity, both labour and total factor productivity. Mainly driven by technological progress, the post 2000 time period report improvements in TFP in all the states. Despite the faster growth in productivity in the post-2000 period, there has been significant variation in labour and total factor productivity across states. We find that differences in infrastructural development at the regional level explain a greater part of the variation in manufacturing productivity growth across Indian states. We find that greater access to power, transport and communication facilities substantially influence total factor productivity at the regional level. Thus our study strengthens the argument (Mitra et al., 2002) that targeting public investment on infrastructures that favour the convergence of industrial productivity constitutes an important element of a strategy of balanced regional growth in India.

## Endnotes

^a^ See Balakrishnan 2010 for recent discussions.

^b^ A comprehensive set of studies on various aspects of manufacturing sector can be found in Tendulkar et al. 2006.

^c^ Trivedi et al. 2011 provides a review of studies on regional manufacturing performance.

^d^Ray (2002) addressed this aspect but for a shorter time period, from 1986–87 to 1995–96.

^e^ See Goldar and Kumari 2003 for detailed exposition of this argument.

^f^ It should however be noted that non-DEA approaches are no less appealing and can also be estimated without bias. Of late, varying coefficients frontier model is suggested for estimating productive efficiency without bias (See Kalirajan and Obwona, 1994).

^g^ Malmquist productivity indexes were first introduced into the literature by Caves et al. (1982) and were empirically applied by Fare et al. (1994). FGNZ developed a non-parametric approach for estimating the Malmquist indexes, and showed that the component distance function could be derived using a DEA-like linear program method. They also decomposed total factor productivity indexes into efficiency change and technical change components. According to them, the total factor productivity may grow by more efficient utilization of resources and/or by technical change.

^h^ An important issue that has to be addressed while measuring TFP growth is the returns to scale properties of the technology in use. Following Grifell-Tatje and Lovell (1995) we use CRS technology. For estimation, we have used DEAP 2.1, a program for data envelopment analysis developed by Coelli (1996).

^i^ Considerations of consistency of the data base for the entire time period restrict us to limit to fifteen states.

^j^ While we are aware of the bias caused by the single deflation procedure and the superiority of double deflation, arriving input price deflators across states runs into difficulty due to lack of availability of appropriate price data and input–output tables. Hence we prefer single deflation as a second best alternative.

^k^ There have been a number of studies in the Indian context too testing for growth and productivity convergence and the results have been mixed. But most of these convergence studies are for the overall economy see Rao et al. (1999); Ahluwalia (2002) and Nagaraj et al. (2000).

^l^ Ark et al. 2009 provides the following explanation for lack of labour productivity convergence across states in Indian manufacturing sector: “This suggests that the kind of market forces that have led to the alignment of unit labour costs (ULC) across provinces in China are not at play (yet) in the case of India and points to the immobility of resources across space and industries”.

^m^ The estimation procedure is presented in Appendix I.

^n^ If we estimate the convergence function for the post-1990 period excluding the state of Assam, treating it as an outlier, then the divergence in labour productivity appear much starker.

^o^ Much of the previous literature on this aspect analyzed the impact by constructing an index of infrastructure. One limitation of this approach is that it would be difficult to disentangle the role of each infrastructural variable on growth. Our approach would help us to understand the importance of these variables at the regional level as the progress of these variables varies widely across Indian states.

^p^ We also included gross fixed capital formation and credit-deposit ratio as independent variables but did not yield significant results.

^q^ The coefficient of RDDEN is significant between 10 and 15 per cent.

## Appendix I: labour productivity convergence

The dependent variable is average labour productivity growth over a specified time period and the logarithm of initial labour productivity is the only regressor. This kind of estimation is referred to as Barro-regressions in the literature after Barro (1991). More specifically, the model to be estimated is

The average labour productivity growth rate is the term on the left hand side, α and β are the parameters to be estimated, and *ε*_*i*_ is an error term. If the estimated β is negative, we conclude that the data exhibits absolute beta-convergence.
